# Supplementary material for: Intratumoral core microbiota predicts prognosis and therapeutic response in gastrointestinal cancers
Source: Microbiol Spectr. 2025 Aug 28;13(10):e00390-25. doi: 10.1128/spectrum.00390-25 (PMC12502792; doi:10.1128/spectrum.00390-25)
Supplement: Supplemental figures — Fig. S1 and S2. [file spectrum.00390-25-s0001.docx]

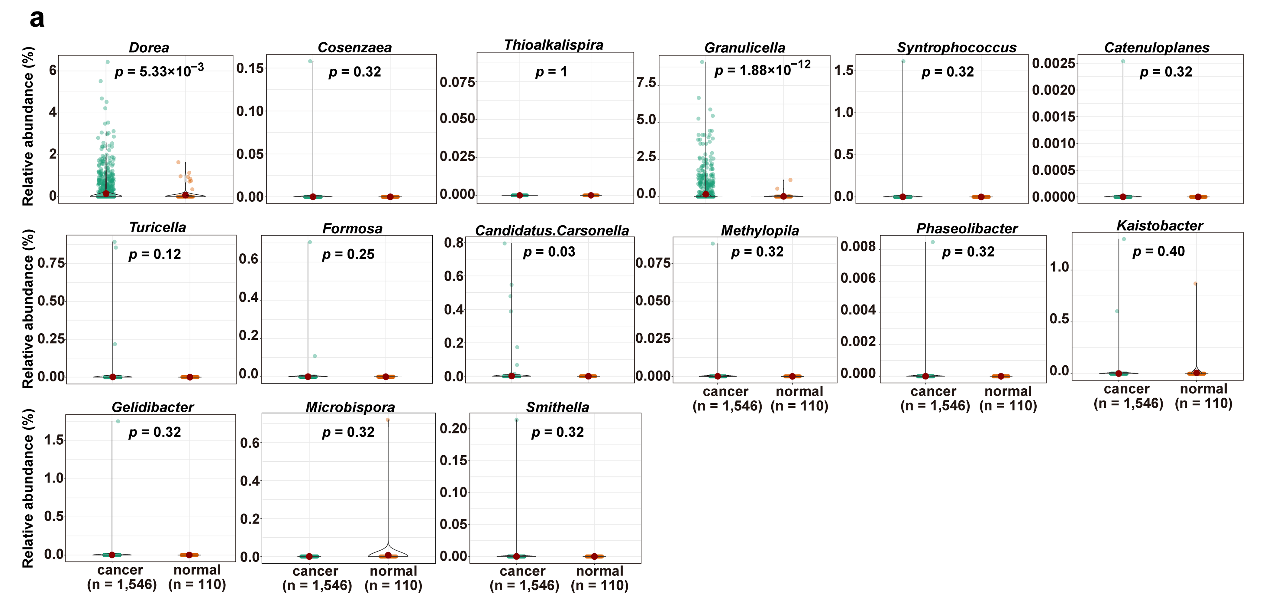


**sFigure 1 (a) Abundance of the 15 genera including in the prognostic model in both cancer and normal groups.**


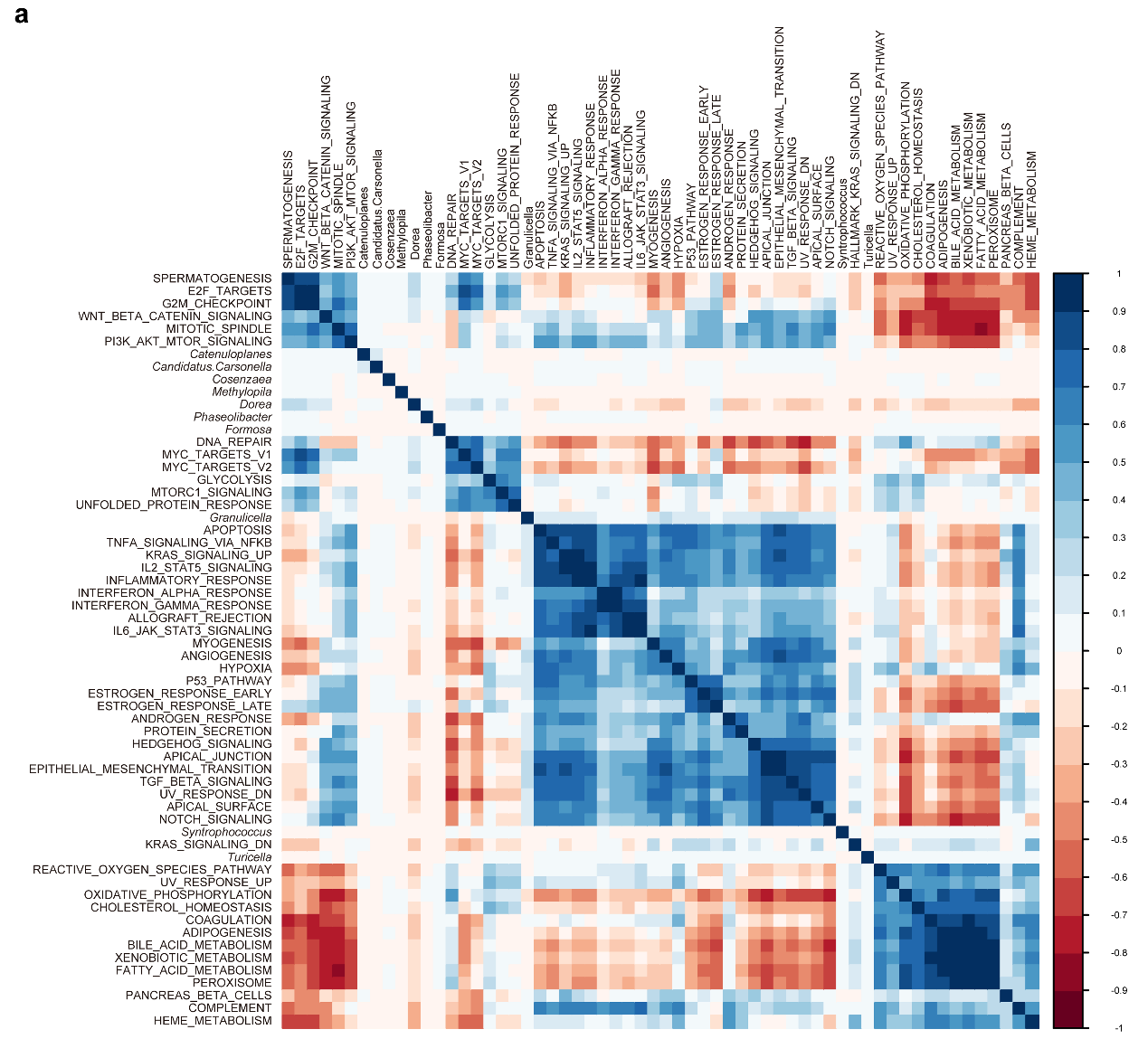


**sFigure 2 (a) Heatmap showing the correlation between the abundance of the 15 genera and the enrichment scores of immunotherapy-predicted pathways.**
