# Supplementary figures and images for: Intratumoral core microbiota predicts prognosis and therapeutic response in gastrointestinal cancers
Source: Microbiol Spectr. 2025 Aug 28;13(10):e00390-25. doi: 10.1128/spectrum.00390-25 (PMC12502792; doi:10.1128/spectrum.00390-25)

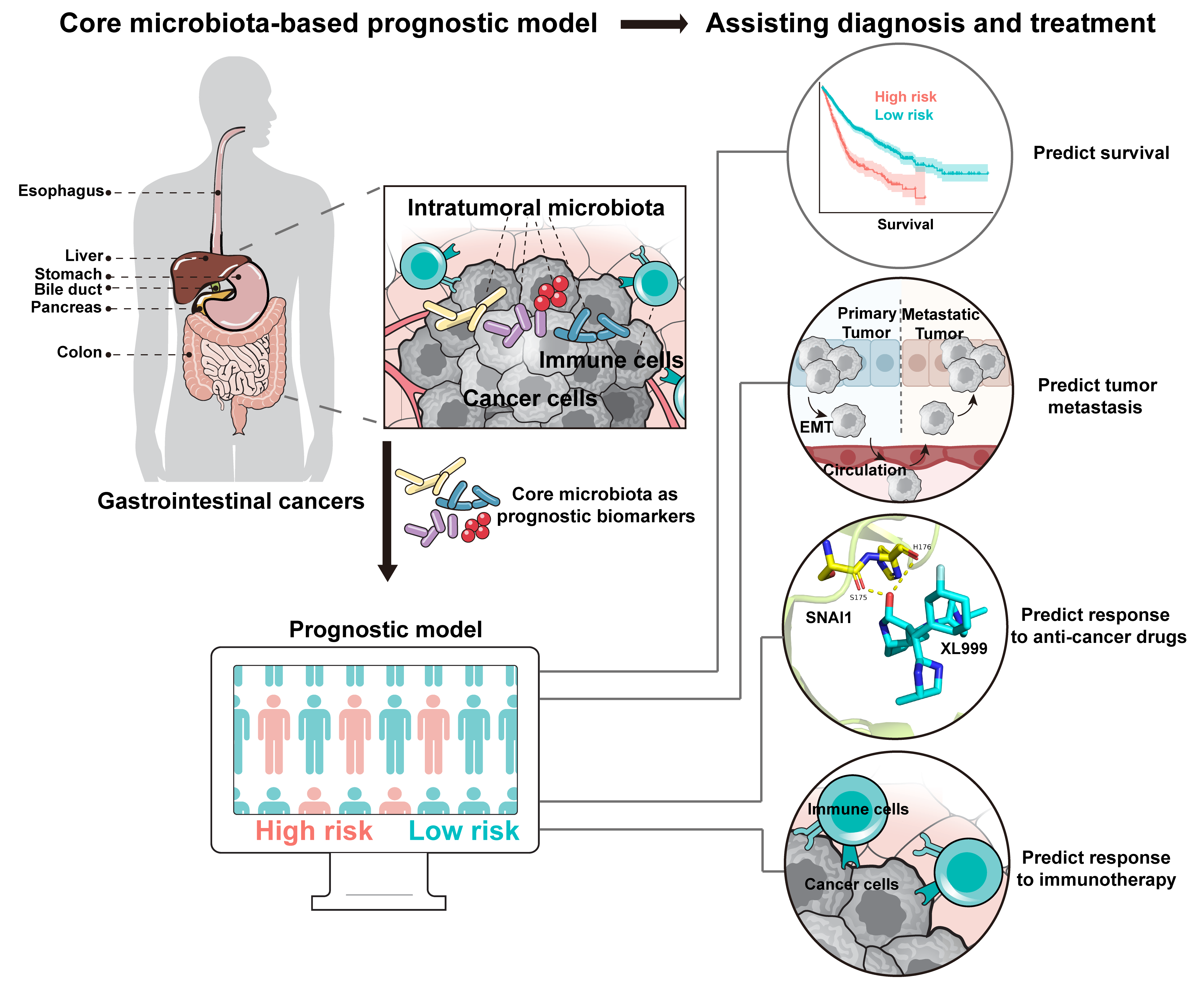

Supplement: Graphic abstract [file spectrum.00390-25-s0002.tif]
